# Supplementary material for: Netcombin: An algorithm for constructing optimal phylogenetic network from rooted triplets
Source: PLoS One. 2020 Sep 18;15(9):e0227842. doi: 10.1371/journal.pone.0227842 (PMC7500971; doi:10.1371/journal.pone.0227842)
Supplement: S4 File — (ZIP) [file pone.0227842.s004.zip › SequenceData-Triplet/sequenceData-10-20-30-0-Triplet/length 300, numseq 30, rate 10^-7/10 seq.pdf]

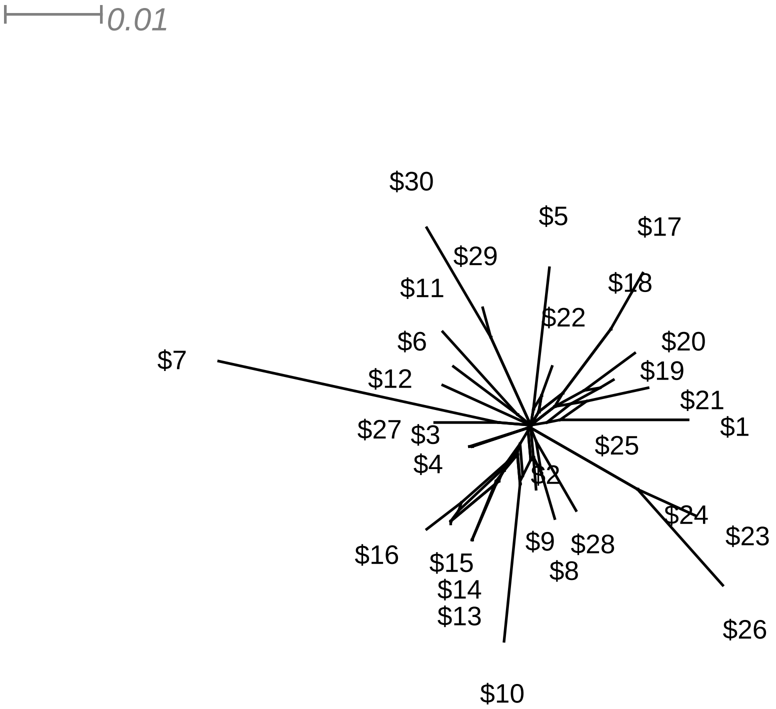
0.01
$30
$5
$17
$29
$18
$11
$22
$6
$20
$7
$19
$12
$21
$27
$3
$1
$25
$4
$2
$24
$23
$9
$28
$16
$15
$8
$14
$13
$26
$10
